# Investigating the coupling effects of rainfall intensity and slope inclination on soil-rock mixture slope stability and failure modes

**DOI:** 10.1371/journal.pone.0314752

**Published:** 2025-02-28

**Authors:** Fanyi Zhou, Hengbin Wu, Yue Qiang, Gang Liu, Zhongxu Zhang, Yi Zhang, Nanxi Chen

**Affiliations:** Civil Engineering College, Chongqing Three Gorges University, Wanzhou, Chongqing, China; Guizhou University, CHINA

## Abstract

The primary factor influencing slope stability is the variation of internal mechanics within the soil-rock mixture caused by rainfall infiltration. Most existing research has focused on how rock content affects the failure of soil-rock mixture slopes. However, there has been insufficient investigation into the coupling effects of rainfall intensity and slope inclination on the stability of soil-rock mixture slopes. Therefore, the model test of soil-rock mixture slope was carried out. The coupling effects of rainfall intensity and slope inclination on water content, earth pressure, pore water pressure, and failure mode of soil-rock mixture slope were analyzed. The failure mode of soil-rock mixture slope induced by rainfall was revealed. The results indicated that an increase in rainfall intensity and slope inclination significantly contributed to the instability of soil-rock mixture slopes and the loss of fine particles. Additionally, the maximum values of water content, earth pressure, and pore water pressure increased progressively. Considering the two influencing factors of rainfall intensity and slope inclination, the calculation formulas related to the fine particle content, maximum water content, maximum earth pressure, and maximum pore water pressure of soil-rock mixture slope were established. The findings of this research provided theoretical support for the construction of soil-rock mixture slopes and the prevention and control of landslide disasters.

## 1. Introduction

Soil-rock mixture landslides are significant geological disasters in the mountainous regions of southwest China, characterized by their large scale and considerable impact [1]. The causes of soil-rock mixture landslides are complicated. Among the various influencing factors, hydraulic influences (rainfall and bank slope erosion) are primary contributors to the instability of soil-rock mixture slopes [2,3]. Recent statistics indicate that rainfall-induced landslide disasters account for 70.5% of geological disasters in China, which include collapses, slides, and flows. In soil-rock mixtures, fine particles migrate and are lost due to hydraulic action, resulting to changes in the internal structure of the soil [4]. This process can significantly compromise slope stability, potentially resulting in landslides [5]. Consequently, it is crucial to investigate the instability mechanisms of soil-rock mixture slopes to develop effective landslide prevention strategies during rainfall.

Model testing is an effective method for investigating rainfall-induced landslides. This approach allows for precise control of boundary conditions and facilitates the observation and monitoring of infiltration, deformation, and failure processes in the slope during rainfall [6]. Numerous studies have elucidated the instability mechanisms of soil-rock mixture slopes through model testing. Mao et al. [7] investigated the particle erosion characteristics and structural evolution mechanisms of soil-rock mixtures under seepage conditions, finding that the loss of fine particles is the primary cause of slope instability in soil-rock mixtures. The stability of soil-rock mixture slopes increases as the loss of fine particles decreases [8]. Fu et al. [9] found that the permeability coefficient of soil-rock mixtures initially decreases and subsequently increases with rising rock content. Furthermore, an increase in the rainfall infiltration angle effectively mitigates the reduction of matrix suction in the sample. Wang et al. [10] discovered that the loss of fine particles and deposition in soil-rock mixtures increases with rising rainfall intensity. Zhang et al. [11] investigated the hydrological response characteristics of soil-rock mixture slopes under rainfall conditions, finding that the matrix suction of soil-rock mixtures significantly decreases with prolonged rainfall.

A large number of studies have examined the effects of rainfall intensity and slope inclination on landslides. Regarding the impact of rainfall intensity on slope stability, Zhou et al. [12] found that continuous rainstorms caused deformation in the upper shallow soil slope of the landslide, accelerating the infiltration of rainwater into the landslide. Li et al. [13] found that as rainfall intensity increases, the water content, earth pressure, and pore water pressure within the slope also increase. Zong et al. [14] reported that rainfall intensity significantly affects the movement and stress levels within the slope. Higher rainfall intensity results in greater displacement at the slope’s base and a wider area of stress influence. The results of Li et al. [15] indicated that with continuous rainfall, pore water pressure initially increased sharply, stabilized, and ultimately exhibited a logarithmic trend. Furthermore, the numerical simulation results by Ng et al. [16] showed that the factor of safety for a vegetated slope under advanced and bimodal rainfall patterns was nearly 53% lower than that under the delayed rainfall pattern. Additionally, the advanced rainfall pattern resulted in the lowest factor of safety. Rainfall-induced soil-rock mixture landslides are complex processes influenced by various internal and external factors, including particle composition and rainfall intensity [17–20]. Regarding the influence of slope inclination on stability, Gallage et al. [21] demonstrated that increasing the slope inclination made it more susceptible to sudden collapse during rainfall. Sun et al. [22] found that the critical threshold for rainfall-induced landslides decreases with increasing slope inclination. A large number of studies have been carried out to analyze the effect of rainfall intensity and slope gradient on soil landslides. However, unlike soil materials, soil-rock mixtures exhibit a looser structure, poorer particle gradation [23], high porosity, high water permeability, and lower stability. Consequently, slopes composed of soil-rock mixtures are more susceptible to plastic damage or slip-pull cracking [5]. The effects of rainfall intensity and slope inclination on the instability process of soil slopes are not easily applicable to soil-rock mixture slopes.

Thus, most researchers have primarily studied the influence of rock content on the instability process and failure modes of soil-rock mixture slopes [6–10]. However, soil-rock mixture landslides are significant and unpredictable disasters [1]. Rainfall intensity and slope inclination are critical factors influencing the slope instability process [12–14,21,22]. However, there are few studies that address the coupled effect of rainfall intensity and slope inclination on the instability of soil-rock mixture slopes. Therefore, it is essential to investigate the coupling effects of rainfall intensity and slope inclination on soil-rock mixture slope stability and failure modes. In this study, a typical soil-rock mixture landslide in the Three Gorges Reservoir area was taken as the object, and a model test of the soil-rock mixture slope was conducted. The failure mode of soil-rock mixture slopes under the coupling effects of rainfall intensity and slope inclination was revealed. The water content, earth pressure, and pore water pressure of the slope were monitored during the landslide process. The research findings provided theoretical support for the construction of soil-rock mixture slopes, as well as for the prevention and control of landslide geological disasters.

## 2. Model test design

### 2.1. Test scheme

Based on soil-rock mixture slope engineering and rainfall data from the Three Gorges area, the model slope dimensions and rainfall intensity were determined. Findings from the investigation of soil-rock mixture landslides in this region indicated that the slope inclination range for these landslides was between 25° and 45°. Consequently, the slope inclinations were set to 30° and 40° in this study. Existing research typically employs a percentile-based threshold to define extreme events. Following Jiang et al. [24], this study utilized three percentile values (85%, 95%, and 99%) as thresholds. The rainfall intensity during the rainy season (June-August) in the Three Gorges Reservoir area was categorized as moderate rain (23.60 mm/h), heavy rain (34.20 mm/h), and heavy rainstorm (58.70 mm/h). The test scheme is presented in Table 1.

**Table 1:** Test scheme.

Working condition	Rainfall intensity (mm/h)	Slope inclination (°)
G1	23.60	30
G2	34.20	30
G3	58.70	30
G4	23.60	40
G5	34.20	40
G6	58.70	40

### 2.2. Slope simulate

**Fig 1:**
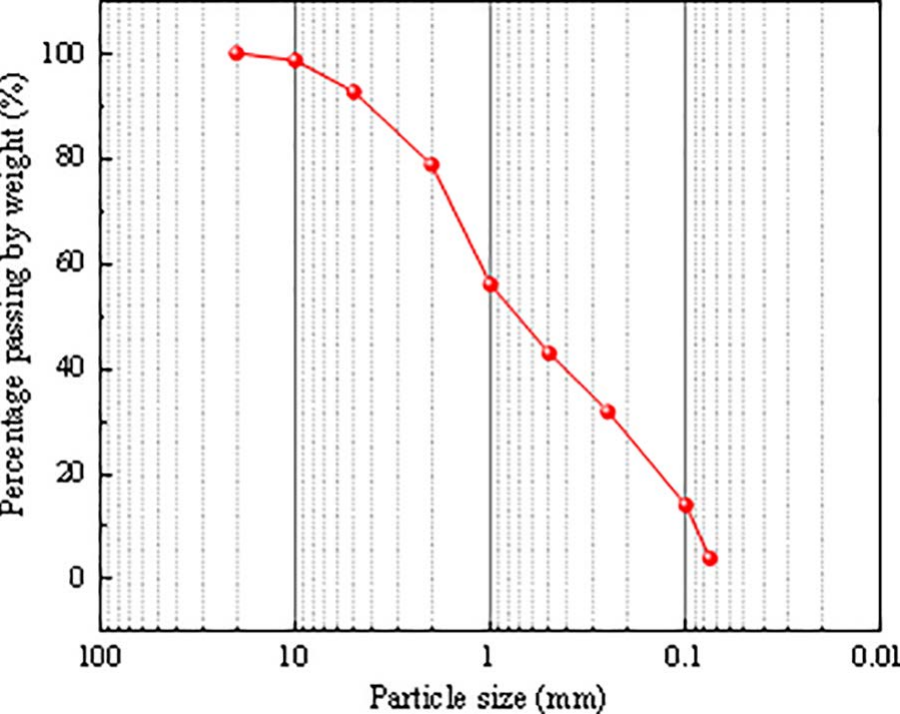
Particle size distribution of soil-rock mixture.

The soil samples used in the slope model were obtained from a soil-rock mixture slope project in the Three Gorges Reservoir area (permission from the Chongqing Three Gorges Reservoir Slope and Engineering Structure Disaster Prevention and Control Engineering Technology Research Center). The soil-rock mixture consisted of coarse and fine particles, with rock content ranging from 22% to 24% in various areas of the soil-rock mixture slope project. The fine particles were mainly composed of silty clay, with a liquid limit of 27.38% and a plastic limit of 15.21%. The compaction test indicated that the maximum dry density of the soil sample was 1.73 g/cm^3^, with an optimal water content of 15.7%. The particle size distribution test result of the soil-rock mixture was presented in Fig 1. The particle size distribution of the model test soil-rock mixture was determined based on a similarity relationship (similarity ratio of 1:100) [25].

**Fig 2:**
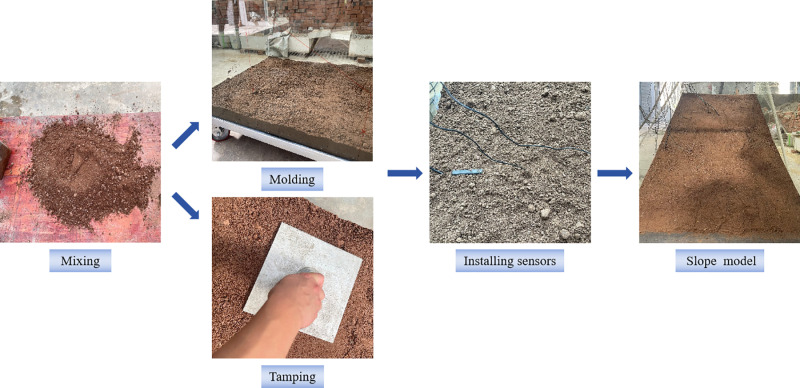
Model preparation process.

The model test is based on an soil-rock mixture slope project in the Three Gorges reservoir area. According to the similarity relationship, the scale of the soil-rock mixture slope was too large to fit into the model box. Due to the complexity of the actual conditions, the model was simplified based on the prototype. The maximum height difference in the landslide area was 60 m, with a slope gradient ranging from 25° to 45°. The slope model was constructed using equal scale, and the similarity ratio with the actual project is 1:100. The modeling process for soil-rock mixture slopes employed a horizontal layered filling method. The specific steps were as follows (Fig 2):

(1) Outlining: Mark the plexiglass on both sides of the model in layers according to the specified slope dimensions.
(2) Layered filling: According to the design of dry density and water content, the mass of soil-rock mixture required for each layer was calculated. The soil-rock mixture was evenly poured into the corresponding layer of the model groove for compaction.
(3) Buried sensors: In order to reduce the interference to the slope, sensors were buried once the filling process reached the designated height. Sensors positioned at the same location were spaced apart to prevent mutual interference.
(4) Adjusting the slope shape: After the slope was fully filled, the surface was level according to the slope contour line. After the model was completed, it was left to stand for 12 hours, and according to the information from the data collector, it was ensured that the slope reached a stable state.

### 2.3. Monitoring system

**Fig 3:**
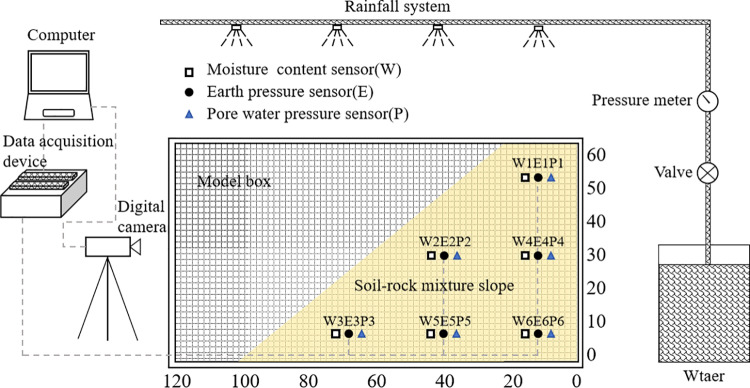
Schematic diagram of the landslide model testing device (units: cm).

The test equipment included the model box, sensor system, acquisition system, and rainfall system (Fig 3).

#### Model box.

The dimensions of the model box were 2 m ×  0.8 m ×  1.2 m (length ×  width ×  height). The three sides were constructed from transparent organic glass, joined by angle steel, while the bottom consists of a 0.2 mm thick impervious high-strength steel plate. All interfaces were sealed with glass adhesive to prevent water leakage.

#### Sensor system.

The sensor system comprised the volumetric water content sensor (precision: ± 3%), earth pressure sensor (precision: ± 0.5%), pore water pressure sensor (precision: ± 0.5%), and a digital camera. The digital camera was installed directly in front of the model box to dynamically monitor the slope’s deformation and failure processes.

#### Acquisition system.

The CR6 acquisition instrument was employed within the data acquisition system. This instrument automatically collected data during the test, providing efficiency and speed.

#### Rainfall system.

The rainfall system was composed of the water tank, water supply valve, pressure gauge, water supply pipe, and low-pressure ultra-fine atomized rainfall sprinkler. The sprinklers were arranged in 2 rows and 3 rows, with a total of 6, and the distance between the two adjacent was 30 cm.

## 3. Analysis of macroscopic deformation characteristics of slope

### 3.1. Failure mode of soil-rock mixture slope

**Fig 4:**
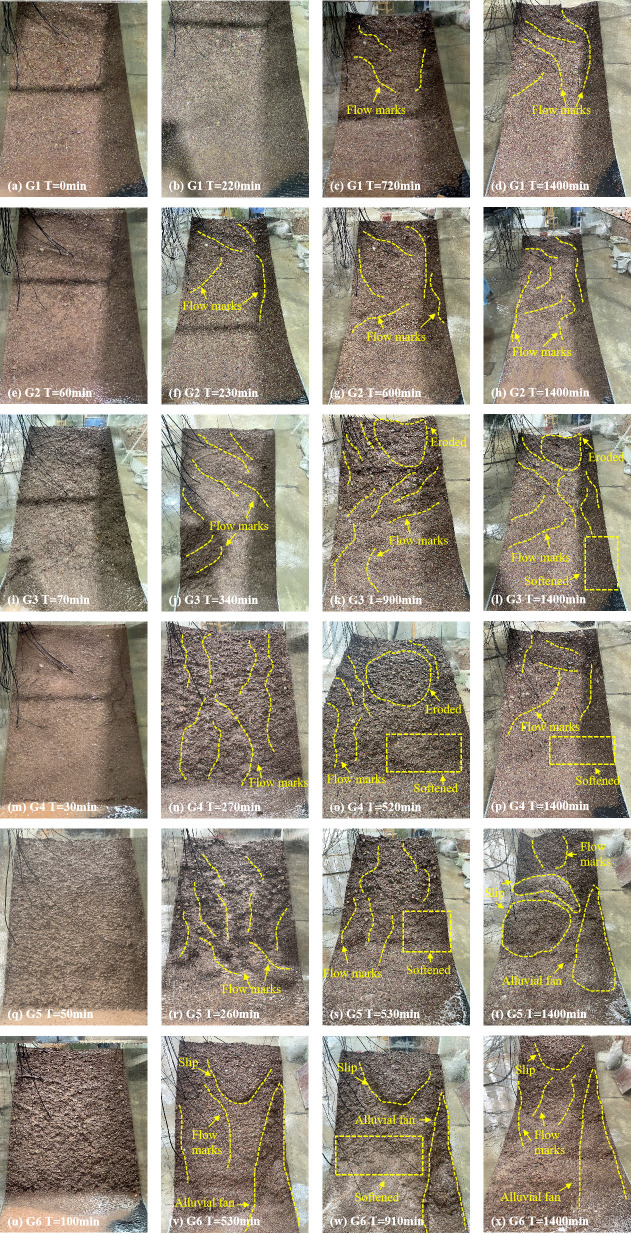
Instability failure process of soil-rock mixture slope.

Fig 4 illustrates the macroscopic deformation of a soil-rock mixture slope induced by rainfall. This deformation and failure mode can be divided into four stages.

#### Erosion stage.

In the initial phase of rainfall, the infiltration capacity of the slope exceeded the rainfall, resulting in the absence of surface runoff. As rainfall continued, the wetting area expanded, leading to a diminished infiltration capacity of rainwater and the subsequent generation of slope runoff. Rainwater eroded the fine particles on the slope’s surface, gradually forming erosion at the slope’s top.

#### Slope toe softening stage.

The front edge of the slope was a shear stress concentration area. Furthermore, following the erosion of the slope’s rear edge, which forms a gully, rainwater accumulated at the front edge of the slope, causing the soil wetting front to advance downward. The increase in water content and the decrease in matrix suction led to the development of pore water pressure, which in turn reduced the shear strength of the soil. The front edge of the slope became wetter and softer and progressively developed toward the back edge, with the front edge gradually forming a freeing surface.

#### Collapse stage.

Under the combined influence of self-weight and rainfall infiltration, the front edge of the slope progressed to a freeing surface, resulting in local collapse and spalling. From an unsaturated state to a saturated state, the sliding surface softened and produced plastic deformation. Finally, when the water content in the deeper sections of the slope reached its maximum, the plastic zone of the sliding surface was interconnected.

#### Instability failure stage.

With the accumulation of rainwater, the soil-rock mixture slope was divided under the combined effects of bulging cracks, shear cracks, and gullies. Cracks, gullies, and the back wall of the landslide provided dominant infiltration channels for rainwater, and slope runoff was converted into groundwater runoff. Rainwater accumulated within the slope, altering the internal seepage field and generating seepage forces. The generation of seepage forces altered the stress environment, while the accumulation of rainfall increased the weight of the soil, thereby enhancing the sliding force. These factors collaboratively contributed to the acceleration of slope deformation and failure.

### 3.2 . Slope deformation of soil-rock mixture slope

Through a comprehensive comparison of the instability and failure evolution processes of soil-rock mixture slopes under six working conditions (G1, G2, G3, G4, G5, and G6) (Fig 4), it is observed that the increase in rainfall intensity led to more significant instability and failure in soil-rock mixture slopes with identical slope inclination. Using the soil-rock mixture slopes of G1, G2, and G3 as examples, higher rainfall intensity resulted in increased movement at the toe of the slope, and the area affected by rainfall on slope deformation was broader. The final failure stages of the G1, G2, and G3 soil-rock mixture slopes were categorized as erosion stage, erosion stage, and slope toe softening stage (Table 2). Additionally, as the slope inclination increased, the loss of the soil-rock mixture matrix under the same rainfall intensity became more pronounced [26]. Using the G3 and G6 soil-rock mixture slopes as examples, a higher slope resulted in greater accumulation of fine particles at the toe. The final failure stages of the G3 and G6 soil-rock mixture slopes were categorized as the slope toe softening stage and the instability failure stage, respectively. These observations and conclusions were essential for accurately understanding the failure evolution of soil-rock mixture slopes under rainfall conditions.

**Table 2:** Statistical table of failure characteristics.

Working condition	Final failure stage	Coarse grain content (>2mm)				Fine grain content (<2mm)			
		Initial	Slope top	Slope middle	Slope toe	Initial	Slope top	Slope middle	Slope toe
G1	Erosion Stage	21.29%	43.10%	40.97%	25.46%	78.71%	56.90%	59.03%	74.54%
G2	Erosion Stage	21.29%	45.22%	42.55%	36.56%	78.71%	54.78%	57.45%	63.44%
G3	Slope toe softening stage	21.29%	62.54%	57.03%	54.51%	78.71%	37.46%	42.97%	45.49%
G4	Slope toe softening stage	21.29%	48.22%	44.11%	32.94%	78.71%	51.78%	55.89%	67.06%
G5	Collapse stage	21.29%	51.79%	49.59%	43.42%	78.71%	48.21%	50.41%	56.58%
G6	Instability failure stage	21.29%	64.54%	60.76%	57.41%	78.71%	35.46%	39.24%	42.59%

Following rainfall, the surface soil was washed away by rainwater, resulting in the loss of fine particles. Notably, the rock exposure of the slope’s top indicated that this area was particularly susceptible to fine grain loss [27,28]. Consequently, particle content tests were conducted at the top, middle, and toe of the slope, yielding the fine particle content for different positions. Table 2 illustrates the migration of soil particles in the soil-rock mixture slope following rainfall. It can be found that the soil-rock mixture slope had more coarse particles at the slope’s top and more fine particles at the slope’s toe under all working conditions. This is attributed to the continuous rainfall runoff on the slope of the soil-rock mixture. The surface water runoff carried the fine particles at the slope’s top of the soil-rock mixture to the slope’s toe and accumulated at the slope’s toe of the soil-rock mixture [29].

**Fig 5:**
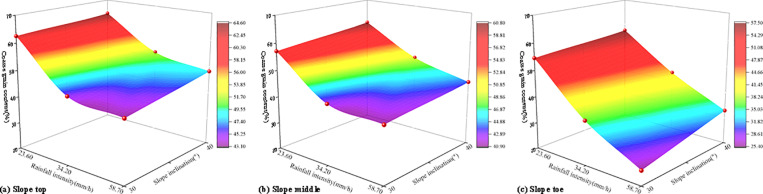
The influence of the coupling effect of rainfall intensity and slope inclination on the coarse grain content.

**Fig 6:**
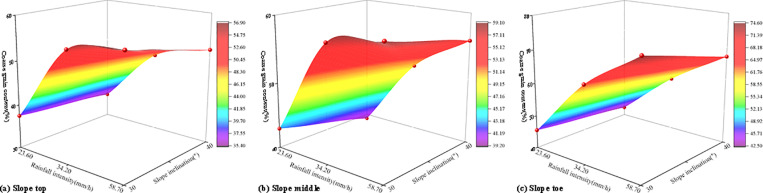
The influence of the coupling effect of rainfall intensity and slope inclination on the fine grain content.

Fig 5 illustrates the influence of the coupling effect of rainfall intensity and slope inclination on the coarse grain content in various sections of the slope. Fig 6 illustrates the influence of the coupling effect of rainfall intensity and slope inclination on the fine grain content in various sections of the slope. It is observed that as rainfall intensity and slope inclination increased, the content of coarse particles in each section of the slope gradually increased, while the content of fine particles gradually decreased. This indicated that as rainfall intensity and slope inclination increased, the loss of fine particles from the soil-rock mixture became more pronounced. Rainfall intensity and slope inclination significantly affect the degree of fine particle loss in soil-rock mixture slopes under rainfall conditions. Based on the test results, under a heavy rainstorm, the degree of fine particle loss in the soil-rock mixture slope at 40° was greater than that in other working conditions. The content of fine particles at the top, middle, and toe of the slope were 35.46%, 39.24%, and 42.59%, respectively.

As shown in Fig 6, the fine particle content of each part of the slope under the coupling of rainfall intensity and slope inclination can be calculated (Table 3).

**Table 3:** Calculation formula of fine particle content in each part of slope.

Soil particle type	sampling position	Formula	R^2^
Fine particle	Slope top	$f_{fine}=0.132\ln\text{RI}\cdot \ln\text{SI}-0.669\ln\text{RI}-0.631\ln\text{SI}+3.438$	0.931
	Slope middle	$f_{fine}=-0.005\ln\text{RI}\cdot \ln\text{SI}-0.164\ln\text{RI}-0.142\ln\text{SI}+1.670$	0.948
	Slope toe	$f_{fine}=0.181\ln\text{RI}\cdot \ln\text{SI}+0.934\ln\text{RI}-0.848\ln\text{SI}+3.642$	0.989

Note: $f_{fine}$ denote the fine grain content (%), RI denote the rainfall intensity (mm/h), SI denote the slope inclination (°), R^2^ denote the coefficient of determination.

## 4. Monitoring data analysis of failure process

### 4.1. Water content of failure process

**Fig 7:**
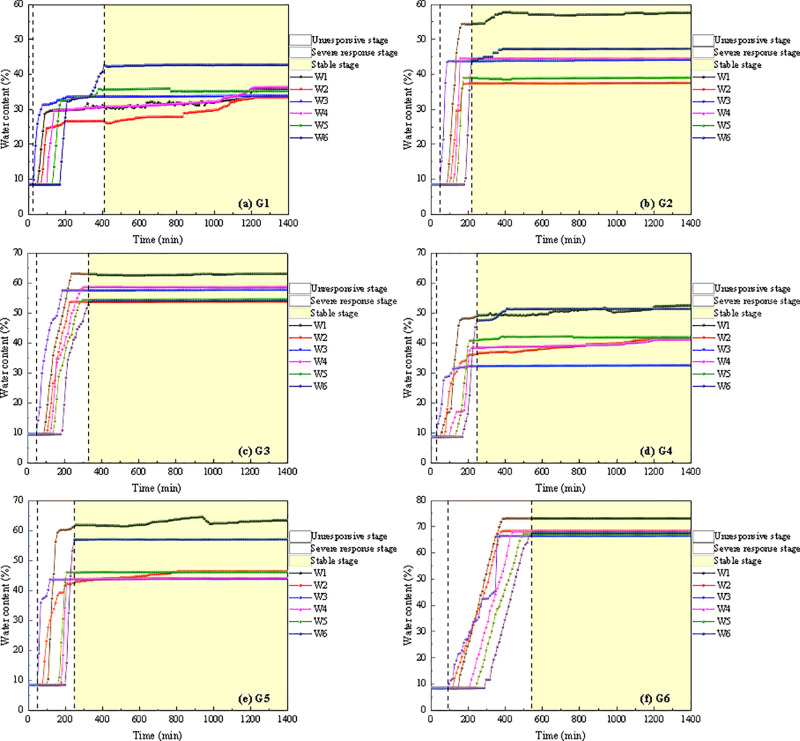
The variation in water content during the instability process of soil-rock mixture slopes.

Fig 7 illustrates the variation in water content during the instability process of soil-rock mixture slopes. The water content at each measuring point remained stable until the rainfall infiltration effect reached the sensor position. With rainfall persisted for a certain duration, the infiltrating water reached the monitoring point, causing a sharp increase in the soil’s water content, which then gradually stabilized. Therefore, the change in water content of the soil-rock mixture slope during rainfall can be divided into three stages: (1) Unresponsive stage. During the initial phase of rainfall, the soil water content remained stable at approximately 9%. (2) Severe response stage. As rainfall persisted, the water content of the soil-rock mixture slope gradually increased, leading to a rapid rise in soil water content. (3) Stable stage. Continuous rainfall caused the soil water content to stabilize.

Fig 7 shows that under all working conditions, the wet peak of rainfall infiltration initially affected the measuring points W1, W2, and W3 on the slope surface. The toe of the slope responded first, followed by the top, and finally the middle, deeper regions of the slope exhibited a slower response speed. The wet peak of rainfall infiltration reached W3 at the toe of the slope at the earliest and W6 inside the slope at the latest. The maximum water content values at various measurement points exhibited distinct stratification corresponding to depth. As depth increased, the maximum water content gradually rose, and the water content at equivalent depths was similar. Furthermore, as rainfall intensity increased, the response rate of slope monitoring points gradually slowed. The study conducted by Li et al. [13] demonstrated that the infiltration capacity of expansive soil slopes varied significantly under different rainfall intensities. As rainfall intensity increased, the response rate of slope monitoring points transitioned from slow to fast. The results of the water content response rate in this study were contrary to the findings of Li et al. [13]. The main reason was that after the soil-rock mixture slope was eroded by rainwater, the lost fine-grained soil was accumulated at the toe of the slope (Table 2). Consequently, the porosity of the soil at the toe of the slope decreased, leading to a reduction in the infiltration rate. Therefore, the infiltration rate was related to the permeability coefficient of the soil-rock mixture. The infiltration of soil-rock mixture slopes was often the result of the coupling of factors such as rainfall intensity, slope inclination and slope failure mode [30].

**Fig 8:**
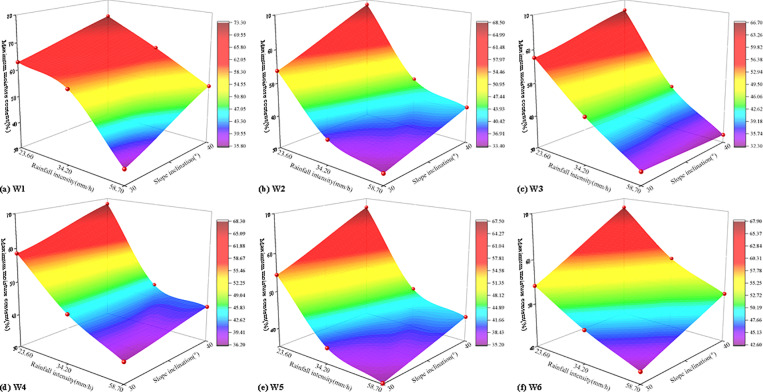
The coupling effects of rainfall intensity and slope inclination on the maximum water content.

The maximum water content of six monitoring points under various working conditions was presented in Table 4. It can be observed that as rainfall intensity and slope inclination increased, the maximum water content at each measuring point gradually rose. This indicated that as rainfall intensity and slope inclination increased, the infiltration of the soil-rock mixture rose. Rainfall intensity and slope inclination were critical factors influencing changes in water content during the instability of soil-rock mixture slopes. Based on the test results, under a heavy rainstorm, the maximum water content at all measuring points on the 40° soil-rock mixture slope exceeded that of other working conditions, with a maximum water content of 73.26%. The coupling effects of rainfall intensity and slope inclination on the maximum water content at each monitoring point were illustrated in Fig 8.

**Table 4:** Maximum water content (%).

Sensor number	Working condition					
	G1	G2	G3	G4	G5	G6
W1	35.81	33.42	33.92	36.29	35.24	42.66
W2	57.69	37.62	44.25	44.59	39.13	47.51
W3	62.92	53.71	57.62	58.51	54.38	54.12
W4	52.39	41.22	32.40	40.99	41.79	51.33
W5	63.44	46.42	43.93	44.13	46.15	57.11
W6	73.26	68.44	66.61	68.25	67.46	67.81

As shown in Fig 8, the maximum water content of each measuring point of the slope under the coupling of rainfall intensity and slope inclination can be calculated (Table 5).

**Table 5:** Calculation formula of the maximum water content.

Sensor number	Formula	R^2^
W1	$w_{\max}=-19.959\ln\text{RI}\cdot \ln\text{SI}+96.182\ln\text{RI}+\text{109.467}\ln\text{SI}-\text{421.733}$	0.919
W2	$w_{\max}=27.287\ln\text{RI}\cdot \ln\text{SI}-69.997\ln\text{RI}-\text{61.623}\ln\text{SI}+\text{169.308}$	0.958
W3	$w_{\max}=41.549\ln\text{RI}\cdot \ln\text{SI}-115.399\ln\text{RI}-\text{150.821}\ln\text{SI}+\text{431.216}$	0.995
W4	$w_{\max}=22.568\ln\text{RI}\cdot \ln\text{SI}-52.271\ln\text{RI}-\text{64.791}\ln\text{SI}+\text{178.946}$	0.942
W5	$w_{\max}=25.967\ln\text{RI}\cdot \ln\text{SI}-66.795\ln\text{RI}-\text{62.307}\ln\text{SI}+\text{177.590}$	0.951
W6	$w_{\max}=19.659\ln\text{RI}\cdot \ln\text{S}-\text{54.310}\ln\text{RI}-\text{33.520}\ln\text{SI}+\text{117.054}$	0.998

Note: $w_{\max}$ denote the maximum water content (%).

### 4.2. Earth pressure of failure process

**Fig 9:**
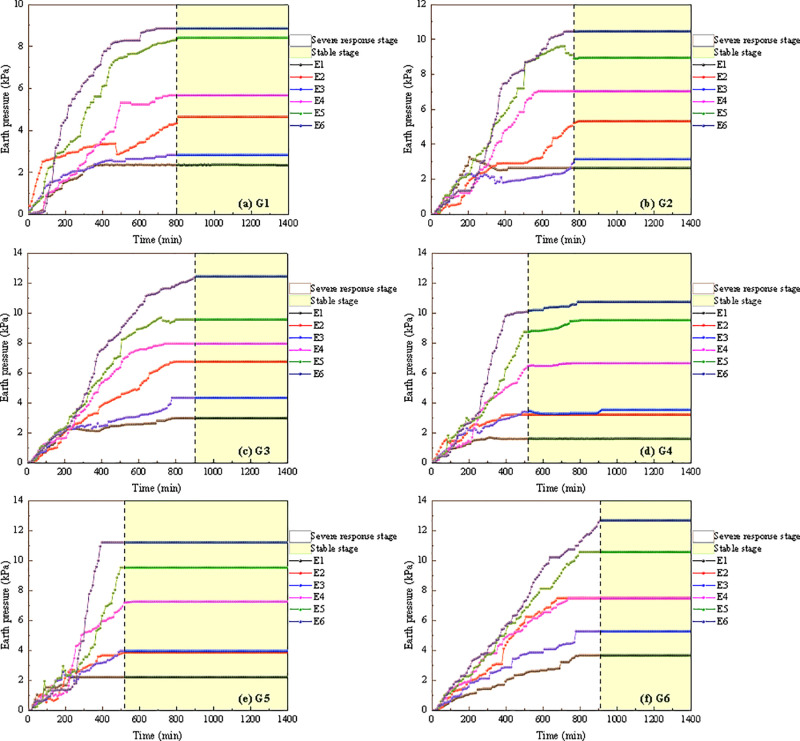
The variation in earth pressure during the instability process of soil-rock mixture slopes.

Fig 9 illustrates the variation in earth pressure during the instability process of soil-rock mixture slopes. It can be observed that earth pressure at each measuring point rose rapidly. However, due to the heterogeneity of the soil, the response time and rate of increase in earth pressure varied across different slope points. This resulted in a complex change in earth pressure within the slope. As rainfall infiltration progressed, the soil was gradually saturated. Under the influence of self-weight stress, the saturated soil gradually consolidated, reducing soil pores, increasing effective stress, and elevating earth pressure at each measuring point. Upon completion of the consolidation process, the earth pressure gradually stabilized. Therefore, the variation in earth pressure of the soil-rock mixture slope during rainfall can be categorized into two stages: the severe response stage and the stable stage. During the severe response stage, the earth pressure at each measuring point exhibited an oscillatory increase. The stress within the slope was primarily transmitted through the rocks, while the fine-grained soil primarily serves as a filler, causing fluctuations in the internal stress of the slope.

With the increase in soil depth, the change in earth pressure also increased (Fig 9). The response speed of earth pressure in shallow soil affected by rainfall was more rapid. Furthermore, the slope’s toe was the exposure of runoff and seepage, rendering it the weakest link in slope stability analysis [31]. The variation in earth pressure within the G2 soil-rock mixture slope became more complex during rainfall, particularly at the measurement points E2 and E3, where earth pressure fluctuated significantly. This is attributed to the friction and rolling of large gravel blocked near the measurement point during slope settlement, resulting in both stress reduction and concentration in the localized area. Moreover, earth pressure at other measurement points fluctuated during the rainfall landslide test, further indicating that the overall stability of the soil-rock mixture slope was significantly affected by rainfall.

**Fig 10:**
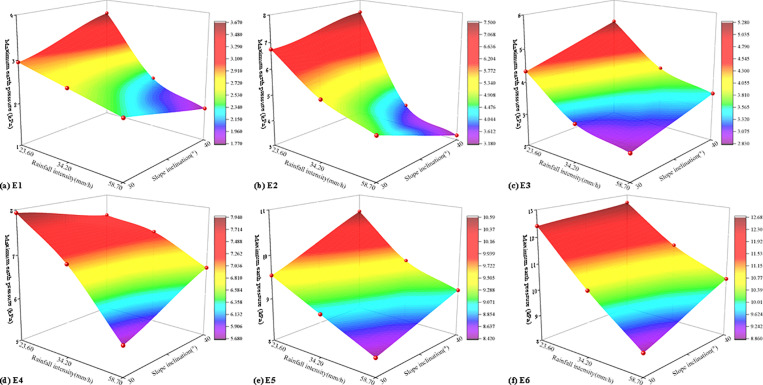
The coupling effects of rainfall intensity and slope inclination on the maximum earth pressure.

The maximum earth pressure of six monitoring points under various working conditions was presented in Table 6. It can be observed that as rainfall intensity and slope inclination increased, the maximum earth pressure at each measuring point gradually rose. Rainfall intensity and slope inclination were critical factors influencing changes in earth pressure during the instability of soil-rock mixture slopes. Based on the test results, under a heavy rainstorm, the maximum earth pressure at all measuring points on the 40° soil-rock mixture slope exceeded that of other working conditions, with a maximum earth pressure of 12.68 kPa. The coupling effects of rainfall intensity and slope inclination on the maximum earth pressure at each monitoring point were illustrated in Fig 10.

**Table 6:** Maximum earth pressure (kPa).

Sensor number	Working condition					
	G1	G2	G3	G4	G5	G6
E1	2.35	4.65	2.83	5.68	8.42	8.87
E2	2.65	5.34	3.16	7.05	8.97	10.48
E3	2.95	6.73	4.32	7.93	9.54	12.41
E4	1.77	3.18	3.51	6.63	9.092	10.27
E5	2.22	3.88	3.97	7.26	9.54	11.21
E6	3.67	7.49	5.28	7.49	10.58	12.68

As shown in Fig 10, the maximum earth pressure of each measuring point of the slope under the coupling of rainfall intensity and slope inclination can be calculated (Table 7).

**Table 7:** Calculation formula of the maximum earth pressure.

Sensor number	Formula	R^2^
E1	$\sigma_{\max}=5.112\ln\text{RI}\cdot \ln\text{SI}-\text{16.735}\ln\text{RI}-\text{18.676}\ln\text{SI}+\text{63.825}$	0.967
E2	$\sigma_{\max}=8.898\ln\text{RI}\cdot \ln\text{SI}-\text{27.956}\ln\text{RI}-\text{34.451}\ln\text{SI}+\text{114.466}$	0.945
E3	$\sigma_{\max}=1.031\ln\text{RI}\cdot \ln\text{SI}-\text{1.828}\ln\text{RI}-\text{0.859}\ln\text{SI}+\text{0.343}$	0.975
E4	$\sigma_{\max}=-5.218\ln\text{RI}\cdot \ln\text{SI}+\text{20.158}\ln\text{RI}+\text{19.557}\ln\text{SI}-\text{68.278}$	0.939
E5	$\sigma_{\max}=1.549\ln\text{RI}\cdot \ln\text{SI}-\text{4.059}\ln\text{RI}-\text{2.908}\ln\text{SI}+\text{14.522}$	0.991
E6	$\sigma_{\max}=-4.219\ln\text{RI}\cdot \ln\text{SI}+\text{18.209}\ln\text{RI}+\text{17.928}\ln\text{SI}-\text{64.242}$	0.998

Note: $\sigma_{\max}$ denote the maximum earth pressure (kPa).

### 4.3. Pore water pressure of failure process

**Fig 11:**
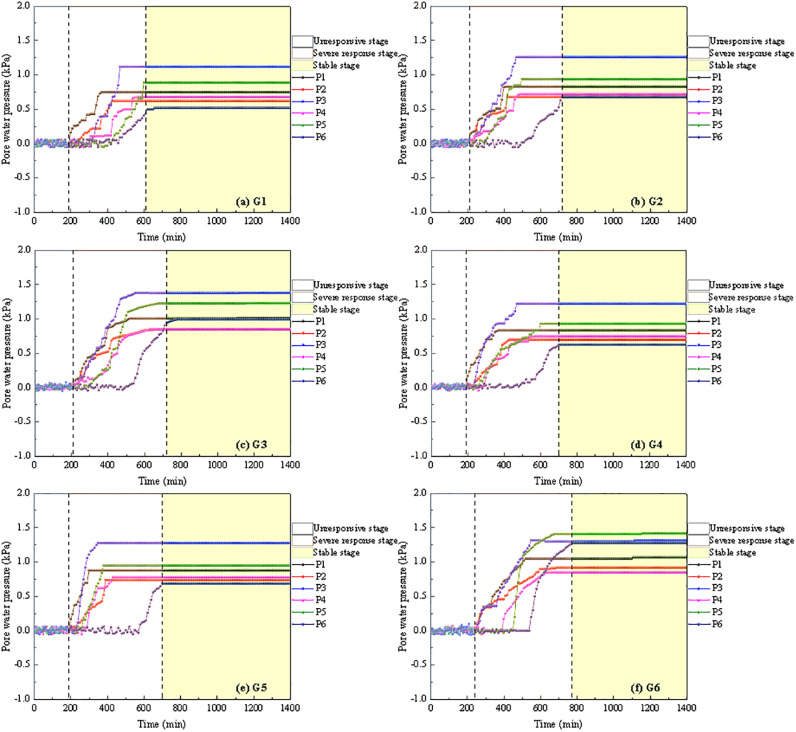
The variation in pore water pressure during the instability process of soil-rock mixture slopes.

Fig 11 illustrates the variation in pore water pressure during the instability process of soil-rock mixture slopes. During the rainfall process, variations in pore water pressure at each measurement point were consistent with the change in water content. The pore water pressure at each measurement point rose rapidly following the initial response and ultimately stabilized. Therefore, the change in pore water pressure of the soil-rock mixture slope during rainfall can be divided into three stages: unresponsive stage, severe response stage, and stable stage. Based on the response time of pore water pressure, the slope surface exhibited the shortest response time. Following rainwater infiltration, pore water pressure began to rise and ultimately reached its maximum. In the interior of the slope, the maximum pore water pressure was relatively low.

Under varying rainfall intensities and slopes, the changes in pore water pressure can be summarized as follows: In the early stage of rainfall infiltration, the growth rate was rapid. As the rainfall continued, the saturation and the saturated areas expanded, leading to a gradual decline in the growth rate. These findings were consistent with the results of Hu et al. [8] regarding the hydrological response of soil-rock mixture slopes under rainfall conditions. From the perspective of pore water pressure increasing, the rise in pore water pressure at the slope’s toe of G1, G2, G3, G4, and G5 soil-rock mixture slopes was greater than that at the slope’s top. In contrast, the increase in pore water pressure within the G6 soil-rock mixture slope exceeded that at the slope’s toe. This phenomenon can be attributed to the significant sliding of the G6 soil-rock mixture slope during rainfall, which caused the slope’s toe sensor to shift along with the matrix, resulting in the P6 sensor relocating to the slope’s toe (Fig 4). By considering the macroscopic deformation characteristics of the slope alongside the changes in volumetric water content, it was evident that cracks form at the top of the G6 soil-rock mixture slope following local sliding. This development created a favorable pathway for rainwater to infiltrate deeper into the slope. Rainwater not only softened the crack boundary but also caused seepage within the crack, leading to deeper fissures. Consequently, the water content of the G6 soil-rock mixture slope increased significantly, resulting in a considerable rise in pore water pressure within the slope [32–34]. The G1, G2, G3, and G4 soil-rock mixture slopes did not generate sufficient slope runoff (Fig 4). In contrast, the G5 and G6 soil-rock mixture slopes were prone to sliding at the top, and the resulting cracks enhanced the slope’s infiltration capacity. Thus, the increase in pore water pressure was closely linked to rainfall intensity and the slope’s infiltration capacity [35].

**Fig 12:**
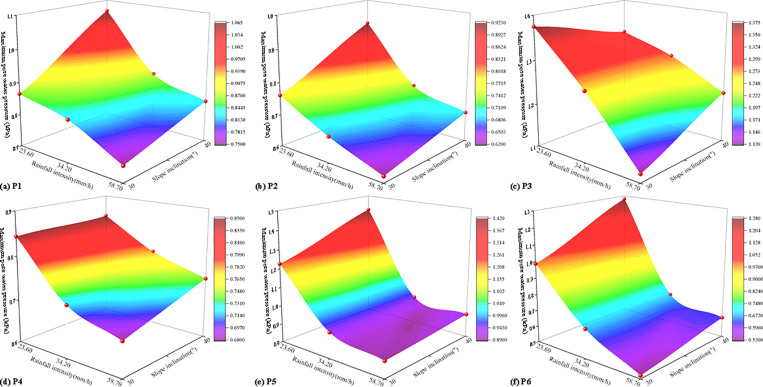
The coupling effects of rainfall intensity and slope inclination on the maximum pore water pressure.

The maximum pore water pressure of six monitoring points under various working conditions was presented in Table 8. It can be observed that as rainfall intensity and slope inclination increased, the maximum pore water pressure at each measuring point gradually rose. This indicated that as rainfall intensity and slope inclination increased, the infiltration of the soil-rock mixture rose. Rainfall intensity and slope inclination were critical factors influencing changes inpore water pressure during the instability of soil-rock mixture slopes. Based on the test results, under a heavy rainstorm, the maximum pore water pressure at all measuring points on the 40° soil-rock mixture slope exceeded that of other working conditions, with a maximum earth pressure of 1.42 kPa. The coupling effects of rainfall intensity and slope inclination on the maximum pore water pressure at each monitoring point were illustrated in Fig 12.

**Table 8:** Maximum pore water pressure (kPa).

Sensor number	Working condition					
	G1	G2	G3	G4	G5	G6
P1	0.750	0.620	1.120	0.680	0.890	0.520
P2	0.830	0.680	1.260	0.720	0.940	0.680
P3	0.865	0.760	1.375	0.844	1.226	0.988
P4	0.828	0.692	1.216	0.740	0.926	0.622
P5	0.880	0.740	1.280	0.780	0.950	0.690
P6	1.065	0.923	1.320	0.850	1.420	1.280

As shown in Fig 12, the maximum pore water pressure of each measuring point of the slope under the coupling of rainfall intensity and slope inclination can be calculated (Table 9).

**Table 9:** Calculation formula of the maximum pore water pressure.

Sensor number	Formula	R^2^
P1	$P_{\max}=0.502\ln\text{RI}\cdot \ln\text{SI}-\text{1.585}\ln\text{RI}-\text{1.420}\ln\text{SI}+\text{5.209}$	0.964
P2	$P_{\max}=0.370\ln\text{RI}\cdot \ln\text{SI}-\text{1.105}\ln\text{RI}-\text{0.986}\ln\text{SI}+\text{3.491}$	0.974
P3	$P_{\max}=-0.569\ln\text{RI}\cdot \ln\text{SI}+\text{2.211}\ln\text{RI}+\text{2.114}\ln\text{SI}-\text{6.924}$	0.970
P4	$P_{\max}=-0.216\ln\text{RI}\cdot \ln\text{SI}+\text{0.919}\ln\text{RI}+\text{0.922}\ln\text{SI}-\text{3.047}$	0.979
P5	$P_{\max}=0.645\ln\text{RI}\cdot \ln\text{SI}-\text{1.813}\ln\text{RI}-\text{2.036}\ln\text{SI}+\text{6.579}$	0.919
P6	$P_{\max}=0.804\ln\text{RI}\cdot \ln\text{SI}-\text{2.217}\ln\text{RI}-\text{2.417}\ln\text{SI}+\text{7.092}$	0.933

Note: $P_{\max}$ denote maximum pore water pressure (kPa).

## 5. Discussion

This study examined the coupling effects of rainfall intensity and slope inclination on soil-rock mixture slopes, investigating both the formation process and destabilization mechanisms of landslides. Rock permeability was lower in soil-rock mixtures compared to soil slopes [36], and this low permeability led to more intense runoff erosion of the slope surface. Additionally, the low permeability of the soil-rock mixture resulted in sensor response times that were nearly double those recorded in the study by Li et al. [13] on expansive soil slopes under rainfall conditions. In addition, the instability of the soil-rock mixture slopes increased with increasing rainfall intensity, which was consistent with findings from previous studies on soil slopes [13–16]. It is noteworthy that under moderate rain (23.60 mm/h), the damage to soil-rock mixture slopes started from the slope’s top, whereas under heavy rain and heavy rainstorm, the damage to slopes started from the foot of the slope. Gallage et al. [21] observed that the time of damage to soil slopes under rainfall decreased with an increase in slope gradient. Similarly, the same phenomenon was observed for soil-rock mixture slopes, and the trend was more pronounced with increasing rainfall intensity. Additionally, the applicability of the formulas established in this study for fine particle content, peak water content, peak earth pressure, and peak pore water pressure to soil-rock mixture slopes under different boundary conditions requires further verification and refinement.

This study aimed to investigate the coupling effects of rainfall intensity and slope inclination on the stability of soil-rock mixture slopes and to reveal the failure mode of soil-rock mixture slopes. However, some spatial variability in soil parameters may exist under field conditions due to complex geological processes and mineral compositions [37]. Meanwhile, the vegetation cover on the slope surface can affect the hydraulic properties of soil-rock mixtures [38,39]. The remoulded soil samples do not completely represent the distribution of the dominant seepage pipe network within the natural soil-rock mixture slope. The damage to natural soil-rock mixture slopes represented a complex physical process resulting from a combination of factors, including particle composition and structure, rainfall, groundwater, and soil anisotropy [40,41]. In subsequent research, the properties of natural soil-rock mixtures can be restored and characterized more comprehensively by further considering factors such as similarity ratio design, slope gradient, vegetation cover, and incorporating centrifuge testing.

## 6. Conclusions

In this study, a typical soil-rock mixture landslide in the Three Gorges Reservoir area was taken as the object, and a model test of the soil-rock mixture slope was conducted. The failure mode of soil-rock mixture slopes under the coupling effects of rainfall intensity and slope inclination was demonstrated. The variation of water content, earth pressure, and pore water pressure in the slope during the process of instability and failure was analyzed. The following conclusions can be drawn:

(1) The failure mode of a soil-rock mixture slope induced by rainfall can be divided into four stages: erosion stage, slope toe softening stage, collapse stage, and instability failure stage. The change in water content and pore water pressure of the soil-rock mixture slope during rainfall can be divided into three stages: unresponsive stage, severe response stage, and stable stage. The change in earth pressure of the soil-rock mixture slope during rainfall can be divided into two stages: severe response stage and stable stage.
(2) As rainfall intensity and slope inclination increased, the failure of soil-rock mixture slope became more pronounced, resulting in greater loss of fine particles. The maximum values of water content, earth pressure, and pore water pressure at each measuring point gradually increased. The fine particle loss of soil-rock mixture slope with rainfall intensity of 58.70 mm/h and slope inclination of 40° ranged from 36.12 to 43.25%, the maximum value of water content was 73.25%, the maximum value of earth pressure was 12.68 kPa, and the maximum value of pore water pressure was 1.42 kPa.
(3) Considering the two influencing factors of rainfall intensity and slope inclination, the calculation formulas related to the fine particle content, maximum water content, earth pressure maximum, and maximum pore water pressure of soil-rock mixture slope were established. The soil-rock mixture slope, with a rock content of nearly 23%, a slope angle ranging from 30° to 40°, and a rainfall intensity ranging from 23.60 mm/h to 58.70 mm/h, allowed for the direct inference of the final failure stage, fine particle loss, maximum water content, maximum earth pressure, and maximum pore water pressure using the calculation formula. This method also reduced the investment of human and material resources.

## Supporting information

S1 Data Minimal data set. (ZIP)
